# Dual Band-Pass Filter Based on Split Ring Resonators with Controlled Asymmetric Bandwidth Response

**DOI:** 10.3390/s26113519

**Published:** 2026-06-02

**Authors:** Patricia Castillo-Araníbar, Alejandro García Lampérez, Daniel Segovia-Vargas

**Affiliations:** 1 Department of Electric and Electronic Engineering, Universidad Católica San Pablo, Arequipa 04001, Peru; 2 Department of Audiovisual Engineering and Communications, Universidad Politécnica de Madrid, 28031 Madrid, Spain; alejandro.garcia.lamperez@upm.es; 3 Department of Signal Theory and Communications, Universidad Carlos III de Madrid, 28911 Getafe, Spain; dansevar@ing.uc3m.es

**Keywords:** microwave, asymmetric response, SRR

## Abstract

A synthesis method for compact dual-band bandpass filters based on split-ring resonators (SRRs) is presented. The method combines coupling-matrix synthesis with an energy-based SRR model with a control technique of the center frequencies and the bandwidth ratio (BWR) of the two passbands. The proposed methodology is experimentally validated for prototypes implemented on Rogers RO3010. Although the synthesis procedure is general in formulation, any change of substrate requires re-optimization of the SRR dimensions, couplings, and achievable bandwidth ratio. Two third-order microstrip prototypes were fabricated on Rogers RO3010 ($\epsilon_{r}=10.2$, h=0.64 mm) to validate the approach. The first prototype operates at 1.9 and 2.4 GHz with measured −3 dB bandwidths of 200 and 100 MHz, insertion losses of 1.0 and 1.95 dB, and BWR ≈ 0.5. The second prototype operates at 1.9 and 2.4 GHz with measured bandwidths of 100 and 200 MHz, insertion losses of 1.8 and 0.6 dB, and BWR ≈ 1.9. The corresponding footprints are 32 × 12.37 ${\mathrm{mm}}^{2}$ and 27.87 × 12.42 ${\mathrm{mm}}^{2}$, respectively. The measured responses agree well with electromagnetic simulations and confirm that asymmetric dual-band bandwidths can be achieved in a compact planar topology without additional reconfigurable elements.

## 1. Introduction

The rapid growth of wireless communication technologies and the coexistence of multiple standards, such as Wi-Fi, WLAN, WiMAX, 3G, 4G, and 5G, have increased the demand for compact and efficient multiband bandpass filters (BPFs). In this context, dual-band BPFs are particularly attractive because they enable channel separation and the simultaneous operation of multiple communication bands within a single microwave front-end. These filters are expected to exhibit low insertion loss, high selectivity, compact size, and tunable or controllable bandwidths to meet increasingly demanding design specifications [1,2,3,4].

Numerous approaches have been proposed to address challenges in dual-band filter design. Advanced miniaturized structures have been explored using coupled lines with stepped impedance stubs [1], CRLH transmission line techniques [2], and resonators with multiple transmission zeros to improve selectivity [3,4]. Although these methods often achieve compactness and improved isolation, they typically involve complex topologies or provide limited flexibility in adjusting the bandwidth of the two bands.

Beyond dual-band designs that mainly control center frequencies and bandwidths, more advanced synthesis-oriented approaches have also been reported. Ref. [5] presented dual-band filters with individually controllable passband responses and orders, where each passband can be assigned a different prototype and order through the element-by-element synthesis of dual-band resonators and inverters. Therefore, although it confirms that independent passband control is possible at the synthesis level, it does not address the specific problem of linking asymmetric bandwidth control to a compact SRR-based planar implementation.

More recently, ref. [6] proposed a multi-band band-pass filter with independently controlled asymmetric dual-band response based on an asymmetric stepped-impedance resonator and a metacell structure. However, the control mechanism is topology-specific and depends on assigning different structural roles to the two passbands, rather than on an analytical dual-band synthesis framework with an explicit bandwidth-ratio parameter. Independent passband tunability has also been demonstrated in compact microstrip dual-band filters; in [7], the authors presented a narrowband dual-band BPF with independently tunable passbands using a compact coupling system integrated with flag-shaped and stepped-impedance resonators. However, the independent control is implemented mainly through geometric retuning of structural parameters, whereas the present work aims to introduce the bandwidth ratio into the synthesis stage itself and relate it to the electromagnetic-energy behavior of SRR-based resonators.

Several works have focused on enhancing dual-band selectivity and miniaturization by introducing transmission zeros and advanced coupling techniques [1,3]. Some have employed complex configurations, such as multi-layer CRLH-TL or dual-mode resonators, to achieve bandwidth tuning [2,8]. However, many of these structures offer fixed or symmetric bandwidths, lack independent control of each band’s fractional bandwidth (FBW), or require complicated feed networks and multilayer integration.

Many technical publications on planar resonant structures use SRRs. An SRR is composed of concentric open conductive rings with gaps placed in opposite positions [9]. A dual-band bandpass filter with a good band-to-band rejection was presented in [10]. In [11], a microstrip filter based on SRRs and DS-SRRs was proposed, where each passband was designed individually through control of quality factors and coupling coefficients. A compact dual-mode dual-band filter with transmission poles in both passbands was introduced in [12], offering low insertion loss through a coplanar-waveguide-fed structure. Additionally, ref. [13] presents a compact dual-band bandpass filter combining DSRRs and irregular SIRs. Independent tuning of each passband is achieved by geometrically adjusting the respective resonators.

SRRs have been recognized as practical elements for compact, multi-resonance filter designs due to their inherent symmetry and strong coupling. Prior work has employed SRRs to implement dual-band filters with symmetrical passbands [14] or to enhance isolation via inductive loading or stub configurations. However, these designs generally offer fixed or symmetric bandwidths and lack direct control over the bandwidth of the two passbands.

Recent dual-band filters have achieved compactness and high selectivity through stepped-impedance stubs, coupled-line structures, and multiple transmission zeros [1,3,4]. Other works have introduced tunability using CRLH-based or folded stepped-impedance resonators [2,8]. However, these studies do not formulate the BWR of the two passbands as an explicit synthesis parameter, and several approaches rely on more complex topologies, multilayer structures, or additional tuning elements [2,8]. In contrast, the present work introduces BWR directly into the synthesis process and links it to the physical SRR design. We propose a novel design methodology for dual-band bandpass filters based on SRRs with controlled asymmetric response. Our approach combines electromagnetic analysis of SRRs with coupling matrix synthesis to enable direct control over both the center frequencies and the BWR. By leveraging the electromagnetic energy stored in the resonator conductors, we establish a mapping between stored energy and the relative bandwidths of each passband. Two prototypes are manufactured to experimentally validate the design, demonstrating flexibility in achieving different BWR values while maintaining consistent return loss and a compact footprint. The proposed method is validated through the design and fabrication of two filter prototypes, whose performance is compared against recent state-of-the-art designs [4,14].

Existing planar dual-band filters generally fall into three categories: designs with fixed or nearly symmetric passband widths, designs that require additional resonators or reconfigurable elements to tune the two bands, and designs that achieve good selectivity at the cost of higher structural complexity. What remains insufficiently addressed is a compact single-layer method that treats the bandwidth ratio between passbands as an explicit synthesis parameter and links that parameter to physical resonator quantities. The present work addresses this gap by combining coupling-matrix synthesis with an energy-based SRR model, thereby enabling analytical control of the two center frequencies and the passband bandwidth ratio within a compact planar topology.

This work makes four main contributions. First, it proposes a unified synthesis-to-implementation methodology for dual-band bandpass filters based on split-ring resonators (SRRs), in which the target specifications ($f_{01},\;f_{02},\;FBW_{1},\;FBW_{2}$) are mapped to a realizable filter network through a coupling-matrix formulation that explicitly incorporates the bandwidth ratio (BWR) as a synthesis parameter. This enables analytical prescription of the asymmetric dual-band response without requiring additional resonators or reconfigurable elements. Second, the method establishes a direct design link between the desired BWR and the electromagnetic behavior of the SRR, using stored magnetic energy and its relationship with susceptance-derivative-based bandwidth metrics to guide the selection of the resonator parameters. Third, by employing concentric SRRs as multi-resonance resonators, the proposed approach reduces the number of design variables, enhances strong internal coupling, and achieves a compact footprint in a single-layer topology. Fourth, the methodology is experimentally validated using two fabricated third-order prototypes that exhibit opposite asymmetric responses while maintaining compact size and consistent center-frequency placement.

With respect to our previous work, the stored-energy-based SRR bandwidth-control concept builds on the controllable dual-band band-stop filter framework reported in [15]. However, the present manuscript extends that concept in three key ways: it targets dual-band bandpass filtering instead of band-stop filtering, it integrates SRR bandwidth-ratio control into a complete coupling-matrix synthesis procedure, and it combines that formulation with the single-to-multiband frequency transformation reported in [16] to provide a full synthesis-to-layout workflow. In addition, the paper contributes new experimental validation through two dual-band bandpass prototypes implemented under the same topology and substrate constraints, which constitutes original material in this manuscript.

The remainder of this paper is organized as follows. Section 2 presents the proposed synthesis method. Section 3 introduces the theoretical analysis of the SRR structure, including the derivation of the BWR parameter, and details the implementation and experimental validation of two dual-band filters with opposite BWR values. Finally, the conclusions and future perspectives are provided in Section 4.

## 2. Dual-Band Pass Filter Design Method

The proposed methodology differs from previous dual-band filter approaches in some aspects. First, the bandwidth ratio (BWR) is introduced as an explicit design parameter in addition to the conventional dual-band specifications of center frequencies and bandwidths. Second, the filter is synthesized through a dual-band coupling matrix whose second-order dual-resonance blocks directly define the resonator-level specifications. Third, these synthesized blocks are mapped onto split-ring resonators (SRRs) by establishing an equivalence between the coupling-matrix model and the physical SRR model. Fourth, the method provides an explicit electromagnetic interpretation of BWR by relating it to the susceptance derivative and to the stored magnetic energy at the two SRR resonances.

The design method uses N/2 coupled SRRs to form a filter; therefore, the total filter order is twice that number, *N*. The whole network will be represented by a coupling matrix with *N* resonances, including input/output couplings, in which N/2 dual-band second-order submatrices can be identified. In this case, dual-band is used as a demonstrative example. Then, for the filter design method, in addition to classical specifications, such as the central frequencies $\left\{\omega_{01},\;\omega_{02}\right\}$, or the resonance bandwidths $\left\{\Delta \omega_{1},\;\Delta \omega_{2}\right\}$, one new design parameter BWR, represented by a number indicating the ratio between the higher resonance bandwidth and the lower resonance bandwidth, is included.

The coupling matrix allows proposing a prototype with any coupling topology based on coupled resonators, which for this proposal are the SRRs. The coupling-matrix synthesis is used to define the SRR’s design parameters. These parameters are the resonant frequencies $\left\{\omega_{r1},\;\omega_{r2}\right\}$ and the already defined BWR, together with the coupling between the rings. The coupling matrix also contains the input and output coupling of the filter [16].

**Figure 1:**
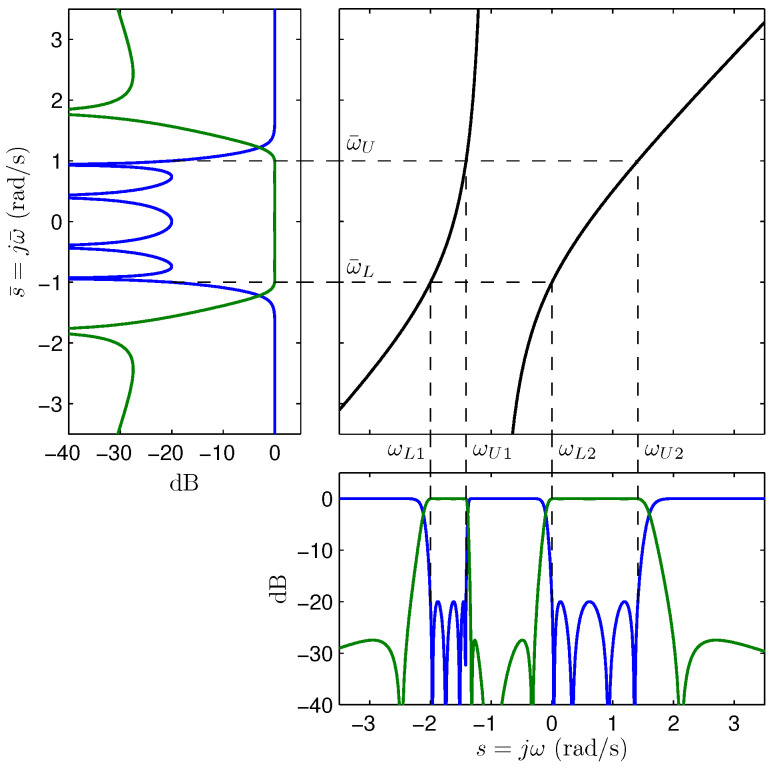
Frequency map between single-band and dual-band prototypes. (Green line $\left|S_{2,1}\right|$, blue line $\left|S_{1,1}\right|$).

A bandpass-to-lowpass frequency transformation is used to convert the dual-band filter specifications into an equivalent single-band prototype response, Figure 1.

**Figure 2:**
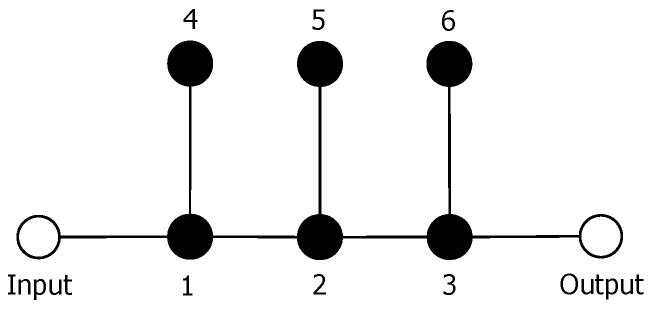
Diagram of nodes and couplings of the dual-band prototype. Lines: couplings, black dots: resonators, white dots: ports. Resonators 4, 5, 6 are the ones attached to the original single-band filter.

Figure 2 illustrates a third-order dual-band filter example. It consists of three primary resonators (1, 2, 3) each attached to an additional resonator (4, 5, 6) to form three dual-resonance blocks. The coupling coefficient, *k*, between each resonator pair and the corresponding susceptance offset, *b* (the low-pass prototype susceptance of the attached resonator), are identical for all the attached resonators.

The common susceptance *b* is computed from the dual-band edge frequencies via (1), and the coupling coefficient *k* is obtained from (2). Although these equations form an overdetermined system, the imposed constraints (which fix $\Delta \bar{\omega }$ and ${\bar{\omega }}_{0}$) ensure a unique solution for *b* and *k*. The input and output coupling values to these dual-resonator blocks are denoted by vectors $m_{S}$ and $m_{L}$, respectively.

(1) $${\bar{\omega }}_{L}=\omega_{L1}+\omega_{L2}+b\;\mathrm{or}\;{\bar{\omega }}_{U}=\omega_{U1}+\omega_{U2}+b.$$

(2) $$\begin{array}{c} {\bar{\omega }}_{L}=\omega_{L1}-\frac{k^{2}}{\omega_{L1}+b}=\omega_{L2}-\frac{k^{2}}{\omega_{L2}+b}\\ {\bar{\omega }}_{U}=\omega_{U1}-\frac{k^{2}}{\omega_{U1}+b}=\omega_{U2}-\frac{k^{2}}{\omega_{U2}+b} \end{array}$$

The next subsections show how to compute the described dual-band coupling matrix, and how its elements are used as specifications to obtain the physical dimensions of the SRRs [15].

### 2.1. Transformation of Second-Order Blocks to SRRs

At this point, the coupling matrix obtained in the previous section represents a network with a coupling topology, as shown in Figure 2. The coupled resonances (1–4), (2–5), and (3–6) represent each SRR, with only one of the resonances interacting with the rest of the circuit (this corresponds to the kI blocks in the coupling matrix). This corresponds to a model of the SRR as two coupled open-loop rings, where only the outer ring is coupled to other elements.

The objective now is to find the equivalence between this model and the one used to obtain the physical dimensions of the SRR, which is based on the two resonances of the whole SRR called $\left\{\omega_{r1},\;\omega_{r2}\right\}$ and the BWR as

(3) $$\mathrm{BWR}≜\frac{\Delta \omega_{r2}}{\Delta \omega_{r1}}.$$

Once the conventional lowpass-to-bandpass transform with the scaling factors already mentioned in Section 2.1 has been applied, each resonator is characterized by the resonant frequencies of the inner and the outer rings as if isolated, $\omega_{1}$ and $\omega_{2}$, respectively, and the coupling coefficient between them *K*. The design process for calculating the SRR specifications [15] is briefly summarized.

The BWR can also be expressed in terms of the susceptance derivative,

(4) $$\mathrm{BWR}≜\frac{B^{\prime }\left(\omega_{r1}\right)}{B^{\prime }\left(\omega_{r2}\right)}.$$

where $B^{\prime }\left(\omega \right)$ is the derivative of the susceptance B(ω), which corresponds to the equivalent lumped-element model of the SRR.

According to [15], the susceptance can be expressed as

(5) $$B\left(\omega \right)=\frac{\omega^{4}-\left({\omega_{1}}^{2}+{\omega_{2}}^{2}+K^{2}Z_{1}Z_{2}\omega_{1}\omega_{2}\right)\omega^{2}+{\omega_{1}}^{2}{\omega_{2}}^{2}}{Z_{1}\omega_{1}\left(\omega^{2}-{\omega_{2}}^{2}\right)\omega }$$

where $Z_{1}$, $Z_{2}$ are the impedance level of each open-loop ring, and $\omega_{1}$, $\omega_{2}$ are the resonant frequencies of each isolated ring.

The solutions of B(ω)=0 are the resonant frequencies of the whole SRR $\left\{\omega_{r1},\;\omega_{r2}\right\}$, while the poles of B(ω), located at $\omega =\{0,\;\omega_{2},\;\infty \}$, are the transmission zeros. This numerator is just a second-degree polynomial in $\omega^{2}$.

**Figure 3:**
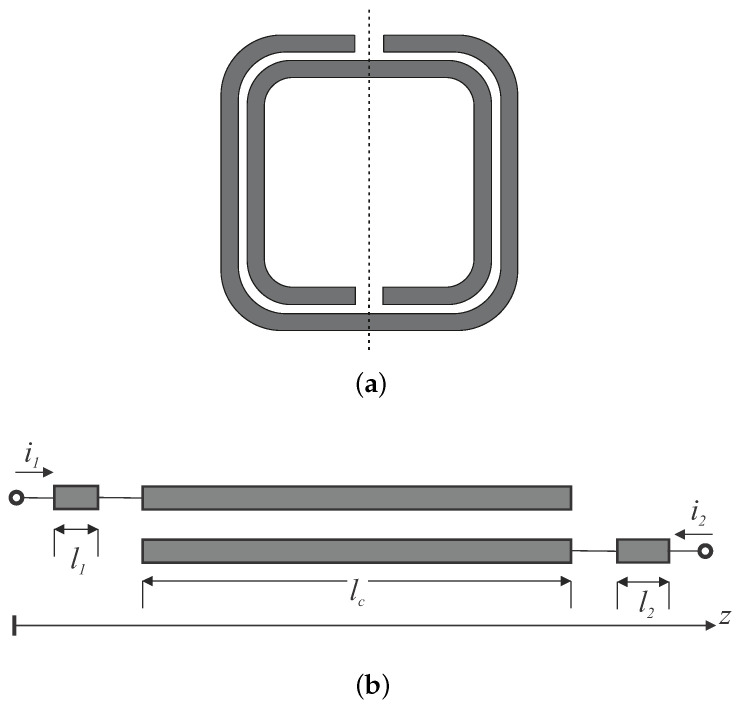
Structure of an SRR (**a**) Layout, showing its symmetry plane, (**b**) equivalent circuit of a half resonator composed of cascaded sections of multi-conductor transmission lines.

Notice that at the resonance frequencies of interest (the first two resonances), the voltage and current distributions in the SRR have odd symmetry with respect to its symmetry plane (Figure 3). See reference [15] by the authors for more details.

Finally, the derivative $B^{\prime }\left(\omega \right)$ can be directly computed from (5):

(6) $$B^{\prime }\left(\omega \right)=\frac{\omega^{2}+\omega_{1}^{2}}{Z_{1}\omega_{1}\;\omega^{2}}+K^{2}Z_{2}\omega_{2}\frac{\omega^{2}+\omega_{2}^{2}}{{\left(\omega^{2}-\omega_{2}^{2}\right)}^{2}}.$$

Thus, these three values $\omega_{r1}$, $\omega_{r2}$, and BWR are calculated from the coupling matrix, and the SRR design method can be readily applied. The process of this method is briefly detailed in Section 2.2. It is worth remarking that the parameter BWR is an expression that relates the resonance bandwidths, as mentioned above. The width of the rings *w* and the separation between the rings *s* are obtained from the calculated 50Ω line and the coupling coefficient, respectively.

### 2.2. SRR Design Method with Controlled BWR

This section summarizes the resonator design procedure. The SRR design method with controlled bandwidth is proposed in [15], where the specifications are the resonance frequencies $\left\{\omega_{r1},\;\omega_{r2}\right\}$ of the resonator and the BWR between the resonances, the parameters *w* and *s*.

It is important to mention that the resonant frequencies $\omega_{r1}$, $\omega_{r2}$, and the coupling factor *k* from the previously calculated coupling matrix are used to design the SRR; the resonance frequencies determine the length of the ring, and the coupling is the distance between them. According to [15], the distance between the rings is an initial parameter to determine the physical dimensions of the SRR. The coupling matrix elements contain the initial values used to design the resonators.

The coupling coefficient associated with the two rings inside each SRR is obtained from the synthesized dual-band block and then implemented physically through the SRR geometry. After the lowpass-to-bandpass transformation, the physical coupling *K* is controlled mainly by the inter-ring spacing *s* and the common coupled length $l_{c}$. A smaller spacing *s* or a larger coupled length $l_{c}$ increases the internal electromagnetic coupling and therefore the resonance splitting. In contrast, the absolute passband bandwidths are governed mainly by the external and inter-resonator couplings, which are set by the feed structure and the spacing between adjacent SRRs.

From the point of view of the design of the dual-band SRRs forming a filter, the BWR, rather than the absolute bandwidths, must be taken into account (the absolute bandwidths are related to the couplings between resonators and input/output couplings). The previously defined BWR is also calculated as the ratio of the stored magnetic energies at the first and second resonances of each resonator [15]:

(7) $$\mathrm{BWR}=\frac{W_{m}\left(\omega_{r1}\right)}{W_{m}\left(\omega_{r2}\right)}.$$

where $W_{m}\left(\omega_{r1}\right)$, $W_{m}\left(\omega_{r2}\right)$ are the corresponding magnetic energies. It can be noticed in (7) that the magnetic energy becomes a measure of the inverse of the bandwidth.

The average magnetic energy stored in a distributed system with multiple conductors [17] is

(8) $$W_{m}=\frac{1}{2}\int i^{T}\left(z\right)L\left(z\right)i\left(z\right)\;dz$$

where L(z) is the matrix of distributed inductances, i(z) the column vector of currents at each conductor and $i^{T}\left(z\right)$ its transpose. Therefore, to obtain the stored magnetic energy, the current distributions along the resonator’s conductors must be calculated at each resonant frequency.

The analysis of an SRR, composed of two tightly coupled rings with open ends located at opposite positions of the rings, can be performed by exploiting its symmetry (see Figure 3a) [15]. A model of a half-resonator is obtained by splitting the SRR along its symmetry plane. Then, the analysis consists of separating the coupled transmission lines into subsections: two single transmission lines and a coupled line section with dimensions of $l_{1}$, $l_{2}$, $l_{c}$, *w*, and *s*, as can be seen in Figure 3b. The analysis of the resonator presented in this work is based on the proposal in [15], where the SRR is modeled as two identical sections of coupled microstrip lines of equal length.

The full current distribution along each conductor is piecewise reconstructed from the current distribution of each section [15].

With the obtained current distribution, the stored magnetic energy can be calculated for each sub-section. The total magnetic energy of the resonator model is

(9) $$W_{m}=W_{m}^{A}+W_{m}^{B}+W_{m}^{C}$$

where $W_{m}^{A}$ and $W_{m}^{C}$ are the energies corresponding to simple transmission lines, while $W_{m}^{B}$ is the energy of the section of coupled lines, according to the sub-sections of Figure 3.

## 3. Dual-Band Bandpass Filter Application

Two prototypes are designed with different BWR values: 0.5 and 2. The specifications are shown in Table 1. The passband locations of the filters correspond to 4G and WLAN systems. For the first prototype, the bandwidth of the lower passband is twice that of the higher one (200 and 100 MHz), whereas for the second prototype, the opposite is true.

The coupling topology of both prototypes is the same as the one in Figure 2. In both cases, the auxiliary single-band network used to build the coupling matrix is a direct-coupled third-order filter with synchronously tuned resonators. Therefore, each filter’s SRRs have an initial model that will vary after EM adjustments, taking into account the input and output couplings.

All the couplings of the filter (input/output couplings, between the rings, and between the SRRs) are computed into the coupling matrix as explained in Section 2. Based on this information, the proposed method provides the physical dimensions needed to obtain the SRR with the required response. The I/O structure can be predicted from the obtained coupling matrix. Notice that the input/output feeds correspond to a single-band filter with a bandwidth equal to the sum of the two bands’ widths in the designed dual-band filter.

The general specifications of a dual-band response can be expressed either as the cutoff frequencies $\left(\omega_{L1},\;\omega_{U1}\right)$, $\left(\omega_{L2},\;\omega_{U2}\right)$, or alternatively, the center frequencies and the bandwidths:

(10) $$\omega_{0i}=\frac{\omega_{Li}+\omega_{Ui}}{2},\;\Delta \omega_{i}=\omega_{Ui}-\omega_{Li},\;i\in \left\{1,2\right\}.$$

Notice that in general, the center frequencies $\left\{\omega_{01},\;\omega_{02}\right\}$ of the dual-band response are not the resonant frequencies $\left\{\omega_{r1},\;\omega_{r2}\right\}$ of the whole SRR, although it is the case for the examples in this section.

Therefore, the resonance frequencies for designing the SRR are directly obtained from the coupling matrix.

An ideal uncoupled resonator exhibits a zero bandwidth at its resonance frequency. The introduction of bandwidth to the resonator is determined by the external quality factor (Q), which governs its coupling with external elements and other resonators. Bandwidth expansion occurs when energy is exchanged with the resonator, a process driven by the external Q. The achievable bandwidth is thus limited by the maximum external Q, which depends on the resonator’s coupling efficiency to its surroundings. When the resonator is strongly coupled, a greater amount of energy is extracted from the system during each cycle, reducing the external quality factor $Q_{\mathrm{ext}}$. Magnetic energy constitutes a significant component of the total stored energy, and the proportion of magnetic energy in the storage process influences the resonator’s ability to retain its energy relative to the amount of energy transferred to the external environment.

### 3.1. Dual-Band Prototype with BWR 1:2

The first step for the filter design is to compute the normalized cutoff frequencies from the specifications, using the conventional bandpass-to-lowpass transform $\omega =\left(f^{2}-f_{0}^{2}\right)/\left(f\Delta f\right)$, where the (arbitrary) normalization parameters are $f_{0}=2.125$ GHz and Δf=0.65 GHz. The resulting cutoff frequencies are $\left\{\omega_{L1},\;\omega_{U1}\right\}=\left\{-1.0903,\;-0.3966\right\}$ and $\left\{\omega_{L2},\;\omega_{U2}\right\}=\left\{0.6592,\;0.9337\right\}$, with a transmission zero located at 0.3598. The third-order transmission zero between the bands is specified in the coupling matrix, which is determined by the resonance frequency of the inner ring. Alternatively, the center frequencies are $\omega_{01}=-0.7435$ and $\omega_{02}=0.7964$, and the bandwidths $\Delta \omega_{1}=0.66936$ and $\Delta \omega_{2}=0.2745$.

**Figure 4:**
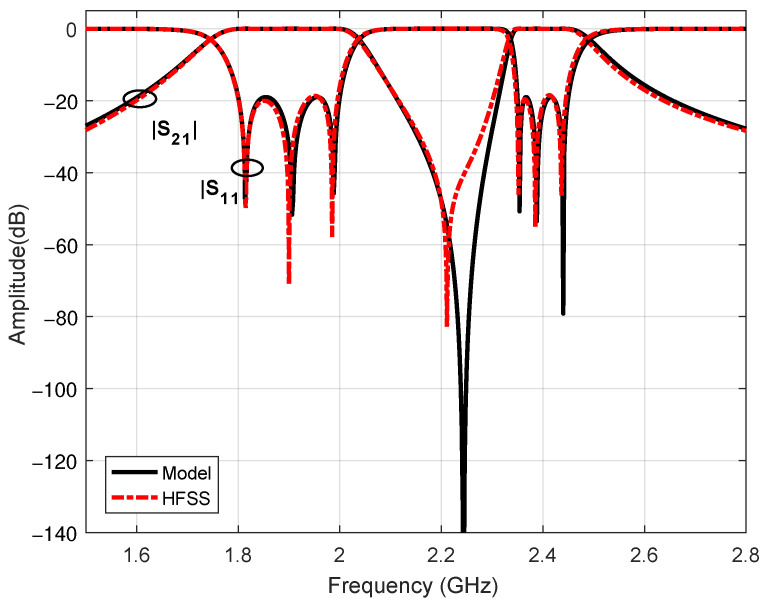
Comparison of frequency response of the prototype with BWR 1:2. Coupling matrix model vs. lossless HFSS simulation.

The single-band cutoff frequencies are $\left\{{\bar{\omega }}_{L},\;{\bar{\omega }}_{U}\right\}=\left\{-0.7909,\;0.1772\right\}$, or equivalently, the center frequency is ${\bar{\omega }}_{0}=-0.3068$ and the bandwidth $\Delta \bar{\omega }=0.9681$. These values are used to synthesize the coupling matrix corresponding to a third-order directly coupled filter (RL=19 dB), which is completed with coupling coefficient k=0.6589 and susceptance b=−0.3598, computed using (1) and (2). The full dual-band coupling matrix is (11). The frequency response of the model is shown in Figure 4, where a comparison between the ideal circuit and electromagnetic model without losses is seen.

The cross-coupling between the inner rings is weak enough to be considered zero valued in the respective positions of the coupling matrix. Otherwise, an additional transmission zero would appear. This is validated with the only transmission zero between the two passbands shown in simulations and in measurements of Figure 4.

The next step is to design each one of the three SRRs. Again, the specifications of the SRR are the resonance frequencies $\left\{\omega_{r1},\;\omega_{r2}\right\}$, the BWR=0.5, and the parameters *w* and *s*. The resonance frequencies of each isolated ring are taken from the calculated coupling matrix. All the resonators have the same resonance frequencies as can be seen in (11). The diagonal coefficients of the coupling matrix become the resonant frequencies of the rings after the lowpass-to-bandpass transformation ($f_{1}=2.028$ GHz, $f_{2}=2.245$ GHz), while the bandpass coupling coefficient is $K=k\;\frac{\Delta f}{f_{0}}=0.2015$.

Finally, the resonant frequencies of the whole SRRs are computed as explained in Section 2.1, resulting in $\left\{f_{r1},\;f_{r2}\right\}=\left\{2.027,\;2.245\right\}$ GHz. In order to fulfill the SRR specifications, (4) and (6) are used to get BWR=0.481. Notice that the non-linear nature of the frequency transform slightly decreases the required BWR (originally 0.5). The unloaded quality factors ($Q_{u}$) of the SRR were extracted by EM simulations, with values of 91 and 94 at 1.9 and 2.4 GHz, respectively.

(11) $$M=\left[\begin{array}{cccccccc} 0 & 0.7341 & 0 & 0 & 0 & 0 & 0 & 0\\ 0.7341 & 0.3068 & 0.4827 & 0 & 0.6589 & 0 & 0 & 0\\ 0 & 0.4827 & 0.3068 & 0.4827 & 0 & 0.6589 & 0 & 0\\ 0 & 0 & 0.4827 & 0.3068 & 0 & 0 & 0.6589 & 0.7341\\ 0 & 0.6589 & 0 & 0 & -0.3598 & 0 & 0 & 0\\ 0 & 0 & 0.6589 & 0 & 0 & -0.3598 & 0 & 0\\ 0 & 0 & 0 & 0.6589 & 0 & 0 & -0.3598 & 0\\ 0 & 0 & 0 & 0.7341 & 0 & 0 & 0 & 0 \end{array}\right]$$

The substrate used to design the microstrip prototypes is Rogers RO3010 with dielectric thickness of h=0.64 mm, relative permittivity $\epsilon_{r}=10.2$ and dielectric loss tan δ=0.0022.

The experimental validation presented in this work is specific to the Rogers RO3010 substrate. Since the effective permittivity, distributed inductance/capacitance, coupling strength, and loss mechanisms depend on the substrate properties, any change of substrate requires re-optimization of the SRR dimensions, coupling distances, unloaded quality factor, and attainable BWR range. Therefore, the results reported here should be interpreted as substrate-specific validations of the proposed design methodology.

The distributed inductances of each section of the SRR (single transmission lines and coupled lines) have been extracted from EM simulations, and their respective values are shown in the first column of Table 2. According to [15], to determine the dimensions of SRRs, certain design parameters are required. The overall open circuit impedance matrix of the half resonator, together with the currents at the symmetry plane and the junctions between sections, is shown in Table 3, at both resonances. The stored magnetic energies at each resonance and the calculated BWR are also shown in Table 3. As stated above, the initial values of ring width and inter-ring spacing are w=0.57 mm and s=0.2 mm, respectively. These values are fine-tuned iteratively to meet the specifications as defined in the method of [15]. From these design parameters, the physical dimensions of the SRR are $l_{1}=1.3$ mm, $l_{2}=0.7$ mm, and $l_{c}=13.1$ mm. The initial SRR design has been changed to account for the input and output coupling adjustments in the EM analysis, resulting in geometric differences between the SRRs. A lossless full-wave simulation was performed using Ansys HFSS 2025 R1 to verify the proposed model. The frequency response is compared to the model frequency response in Figure 4.

**Figure 5:**
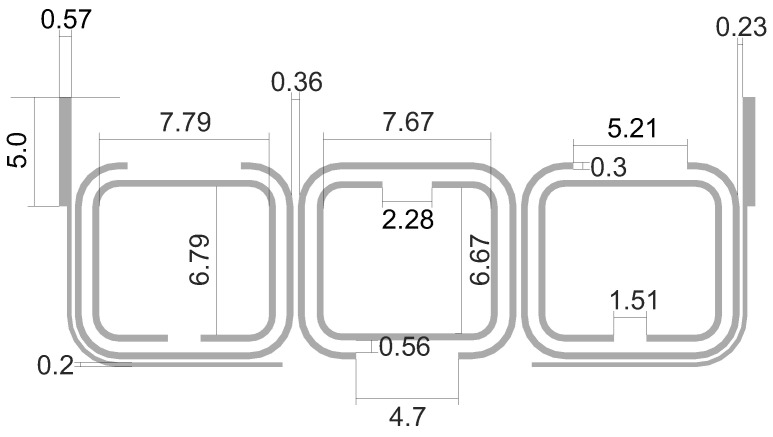
Layout of the dual-band bandpass prototype with BWR 1:2 (dimensions in mm).

**Figure 6:**
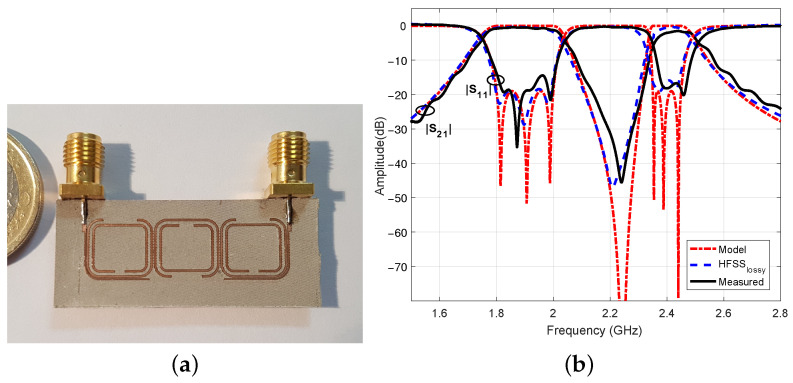
Dual-band bandpass prototype with BWR 1:2. (**a**) Manufactured prototype, (**b**) Measured response (black solid) and simulated response (red dot-line: Ansys HFSS).

The layout of the dual-band filter, resulting from the final adjustment taking into account the whole circuit, is shown in Figure 5. The overall dimensions are $32\times 12.37\;{\mathrm{mm}}^{2}$, and the manufactured prototype is shown in Figure 6a. The simulated and measured scattering parameters of the prototype are shown together with the proposed model in Figure 6b. A comparison between the lossy HFSS model and measurements of the manufactured prototype is shown; the circuit response is included for reference. A full-wave simulation with losses included was performed. Measurements were obtained with an Anritsu VNA Master MS2027C/10. The measured traces were acquired over the 1.5–2.8 GHz range. A full two-port SOLT calibration was applied using a coaxial calibration line, with the reference planes set at 0 mm at both ports and a reference impedance of 50 Ω. No smoothing or aperture post-processing were used in the measurement. The device under test was measured through coaxial connections identified in the instrument file as N-type connectors. The exported traces were then used to compare the measured response with the lossy HFSS simulations and the circuit model, as shown in Figure 6. A comparison of the specifications obtained from simulation and measurements is shown in Table 4. Raw VNA screenshots and exported measured |S11| and |S21| traces for both prototypes are provided as Supplementary Material.

The measured responses exhibit the non-ideal features typically expected in practical microwave measurements and therefore should not be interpreted as perfectly smooth or idealized traces. In particular, the measured results show small ripple effects, frequency shifts with respect to the simulated passbands, a reduction and displacement of the reflection zeros, and an increase in insertion loss relative to the lossy HFSS model. These discrepancies are consistent with the cumulative effect of connector transitions, calibration residuals, fabrication tolerances in narrow-gap regions, and small deviations in the realized coupling dimensions. In the present prototypes, such effects are more evident in the narrowest passband, where the response is intrinsically more sensitive to local perturbations. Therefore, the measured-to-simulated mismatch should be interpreted as a realistic manifestation of practical non-idealities rather than as an inconsistency of the proposed design methodology.

Before making the final prototype, a choice on the manufacturing technology was required. Although laser drilling provides more accurate gap and strip widths, it was found to cause deeper substrate removal around the microstrip lines, which is inconsistent with the assumptions of the proposed design method. For this reason, a photolithographic process was selected. In the prototype with BWR 1:2, the main measurement-to-simulation discrepancy is concentrated in the upper passband, where the narrower bandwidth makes the response more sensitive to small deviations in coupling gaps and strip widths. This explains the larger measured insertion loss in that band and the visible ripple at the upper-frequency side of the response.

A good overall agreement between the circuit model, the lossy HFSS simulation, and the measured response can still be observed in Figure 6b. Nevertheless, the measured response shows a reduction in and small displacement of the reflection zeros, as well as a local increase in $S_{11}$ around 1.97 GHz. These effects are attributed to the combined influence of fabrication tolerances in the narrow feed-coupling regions and the sensitivity of the external coupling to small geometrical deviations. As a consequence, the observed resonant behavior differs slightly from the nominal symmetric assumption used in the initial synthesis stage.

### 3.2. Dual-Band Prototype with BWR 2:1

The specifications for the second prototype are also shown in Table 1; now the upper passband is twice as wide as the lower passband, i.e., BWR=2:1. Since the design procedure is identical to the first prototype, only the results will be briefly stated.

**Figure 7:**
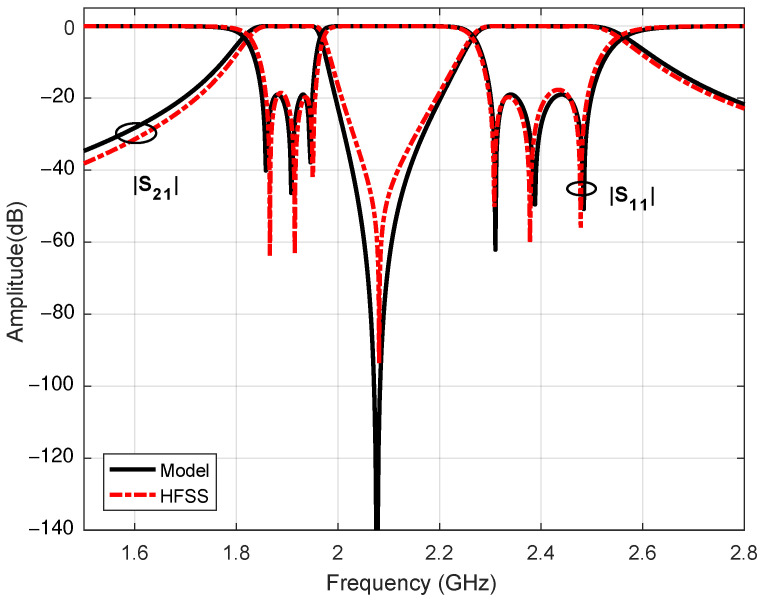
Comparison of frequency response of the prototype with BWR 2:1. Coupling matrix model vs. lossless HFSS simulation.

The lowpass specifications of the network are: the cutoff frequencies $\left\{\omega_{L1},\;\omega_{U1}\right\}=\left\{-1.0878,\;-0.7322\right\}$ and $\left\{\omega_{L2},\;\omega_{U2}\right\}=\left\{0.3742,\;0.9350\right\}$, with the bandpass-to-lowpass transform parameters $f_{0}=2.175$ GHz and Δf=0.65 GHz. The transmission zero is located at −0.3029, the center frequencies are $\omega_{01}=-0.9100$ and $\omega_{02}=0.6546$, and the bandwidths $\Delta \omega_{1}=0.3556$ and $\Delta \omega_{2}=0.5608$. The single-band passband is $\left\{{\bar{\omega }}_{L},\;{\bar{\omega }}_{U}\right\}=\left\{-0.4107,\;0.5057\right\}$, or alternatively, the center frequency is ${\bar{\omega }}_{0}=0.0475$ and the bandwidth $\Delta \bar{\omega }=0.9164$. The resulting coupling coefficient is k=0.7290, and the susceptance b=0.3029. The resulting coupling matrix is (12), with the frequency response in Figure 7.

(12) $$M=\left[\begin{array}{cccccccc} 0 & 0.7142 & 0 & 0 & 0 & 0 & 0 & 0\\ 0.7142 & -0.0475 & 0.4570 & 0 & 0.7290 & 0 & 0 & 0\\ 0 & 0.4570 & -0.0475 & 0.4570 & 0 & 0.7290 & 0 & 0\\ 0 & 0 & 0.4570 & -0.0475 & 0 & 0 & 0.7290 & 0.7142\\ 0 & 0.7290 & 0 & 0 & 0.3029 & 0 & 0 & 0\\ 0 & 0 & 0.7290 & 0 & 0 & 0.3029 & 0 & 0\\ 0 & 0 & 0 & 0.7290 & 0 & 0 & 0.3029 & 0\\ 0 & 0 & 0 & 0.7142 & 0 & 0 & 0 & 0 \end{array}\right]$$

Now, after lowpass-to-bandpass transformation, the resonant frequencies of the inner and outer rings of one SRR are $f_{1}=2.19$ GHz and $f_{2}=2.079$ GHz, respectively, while the bandpass coupling coefficient is K=0.2179. Finally, the resonant frequencies of the whole SRR are $\left\{f_{r1},\;f_{r2}\right\}=\left\{1.908,\;2.386\right\}$ GHz, with BWR=2.019. Again, the SRRs are designed with the same substrate as the previous prototype. The distributed inductances of each section composing the SRRs are included in the last column of Table 2, while the design parameters (impedance matrix, currents and stored magnetic energies at the resonances) are shown in Table 5. The initial value of the width of the rings is w=0.57 mm and the space between the rings is s=0.22 mm. The physical dimensions of the SRR are $l_{1}=1$ mm, $l_{2}=6.5$ mm and $l_{c}=8.89$ mm. The frequency response of the EM lossless simulation is shown in Figure 7.

**Figure 8:**
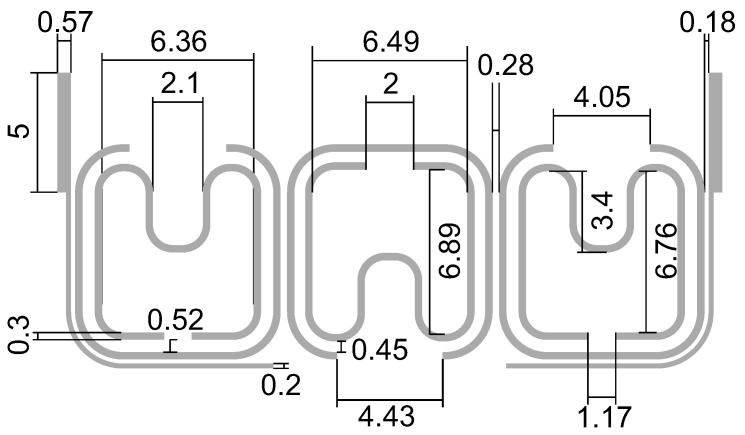
Layout of the dual-band bandpass prototype with BWR 2:1 (dimensions in mm).

**Figure 9:**
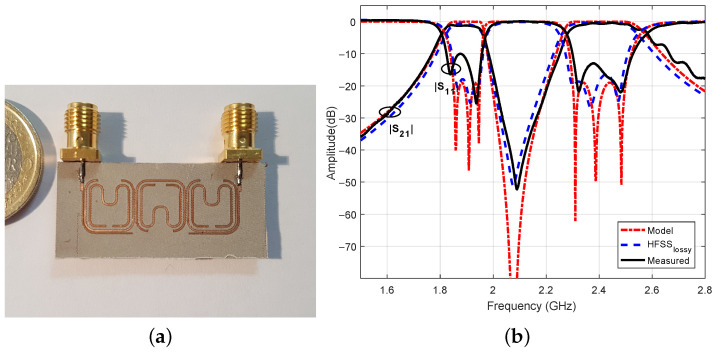
Dual-band bandpass filter with BWR 2:1. (**a**) Manufactured prototype, (**b**) measured response (black solid) and simulated response (red dot-line: Ansys HFSS).

The layout of the filter is shown in Figure 8. The overall footprint is in this case 27.87×12.42 mm^2^, and the manufactured prototype is shown in Figure 9a. Notice that since the inner ring of each SRR is electrically larger than the outer ring, one of its transmission line sections has been meandered. The simulated (losses considered) and measured scattering parameters are presented in Figure 9b with a comparison between simulated and measured results in Table 6. Again, a small reduction in bandwidth occurs due to the presence of losses. In this case, the frequency deviations of the measured response are smaller than in the first prototype. As in the previous prototype, the measured response exhibits practical non-idealities associated with losses, coupling tolerances, and connector/coupling imperfections, although in this case the deviations are less pronounced.

From Figure 9, it can be seen that center reflection zeros have been reduced due to a shift from the imaginary axis in both measured bands. In addition, there is a small increase in the $S_{11}$ parameter at around 1.88 GHz and 2.35 GHz that can be produced due to the fabrication tolerance in the photolithographic process that can hardly reach the required width of 0.2 mm and the spacing of 0.18 mm of the input and output lines.

**Figure 10:**
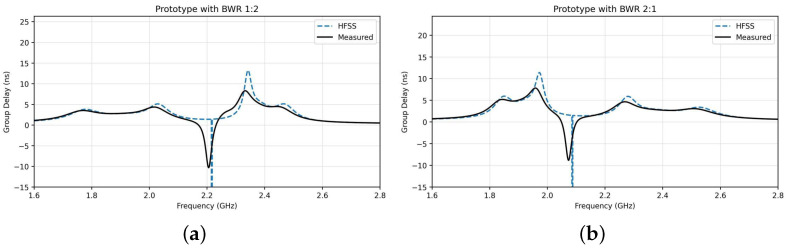
Simulated and measured group delay of the proposed dual-band filters. (**a**) BWR 1:2, (**b**) BWR 2:1 measured response (black solid) and simulated response (blue dashed line: HFSS).

To complement the amplitude-response analysis, the group delay was extracted from the phase of $S_{21}$ for both the simulated and measured responses. The obtained results show reasonably smooth in-band behavior for the two prototypes, with no abrupt excursions inside the useful passbands. From Figure 10, for the prototype with BWR 1:2, the average group delay is approximately 3.1 ns in the lower passband and 4.9 ns (simulation)/4.5 ns (measurement) in the upper passband. For the prototype with BWR 2:1, the corresponding average values are approximately 5.4 ns/5.4 ns in the lower passband and 3.0 ns/2.9 ns in the upper passband. In both filters, the narrower passband exhibits a higher delay and greater in-band variation, whereas the wider passband shows a flatter response. This trend is consistent with the expected phase behavior of narrow dual-band microstrip filters and indicates acceptable phase linearity for the intended 4G/WLAN operation, since the group delay remains bounded and moderately smooth within each passband.

As for the previous resonator, the $Q_{u}$ of this SRR has been extracted for each resonance by EM simulations, with values of 91 and 95 at 1.9 and 2.4 GHz. The geometric parameters of the SRR determine the resonance locations and the $Q_{u}$ of the structures. Thus, the $Q_{u}$ of the SRR has been characterized and compared with other types of resonators used to implement dual-band filters, as can be seen in Table 7. The obtained $Q_{u}$ values of the SRR could be increased by increasing the ring width, but the coupling will decrease as the resonance positions vary.

As shown in Table 7, the unloaded quality factors ($Q_{u}$) of the proposed SRR-based filters are comparable to or slightly higher than those reported in recent literature. Both Filter A (BWR 1:2) and Filter B (BWR 2:1) exhibit $Q_{u}$ values above 90 in both passbands, confirming efficient energy storage and low insertion loss. Compared to loop resonators and CRLH-based structures [2,4], the proposed designs demonstrate improved or equivalent performance without the need for multilayer structures or complex geometries. The values also surpass earlier NB-SRR implementations [14] and match or exceed those of compact stub-loaded resonators [1,3]. These results validate the proposed method’s effectiveness in preserving high $Q_{u}$ while enabling compact layout and controllable bandwidths.

The quality factors reported in Table 7 confirm that the proposed SRR-based filters exhibit comparable or superior $Q_{u}$ values compared to recent designs. Filters using CRLH structures [2] and metamaterial-inspired resonators [1,18] perform well, but often involve more complex topologies. Our design maintains high $Q_{u}$ in both bands with simpler geometry and no multilayer requirements.

For fair comparison, the insertion loss values reported for our prototypes correspond to measured IL at band centers (Table 4 and Table 6), and the fractional bandwidths are computed from measured 3 dB bandwidths.

Table 8 summarizes a comparison with recent dual-band bandpass filters in terms of in-band insertion loss for both passbands, inter-band attenuation, normalized footprint, and fractional bandwidth (FBW). In this context, designs based on multiple transmission zeros can achieve high selectivity, but they do not necessarily provide an explicit and systematic degree of freedom to prescribe the ratio of the bandwidth between passbands. Compared to CRLH/metamaterial-inspired and transmission-zero-based solutions, the proposed SRR-based prototypes maintain a compact planar implementation while achieving low measured insertion loss and strong inter-band rejection, which makes them suitable for multistandard wireless front-ends.

Recent works have demonstrated various dual-band filter solutions with good return loss and miniaturization, such as in [1,3]. However, these designs tend to offer either symmetric bandwidths or limited control over BWR. Others employ reconfigurable elements or multilayer techniques to address tunability [8,19], which increases complexity and fabrication cost. In contrast, the filters presented in this work achieve full analytical control of both FBW and BWR using a single-layer planar topology with SRRs. This feature provides a practical, efficient solution for applications that require asymmetric passbands without compromising performance or simplicity.

A greater fractional bandwidth (FBW) could be achieved; this consideration is contingent on the strongest coupling achievable between the feed lines and the SRRs. External couplings control the overall bandwidth, while internal couplings of the SRR define the resonance positions. Consequently, achieving a larger FBW is dependent on the specific distribution of these couplings.

To emphasize the distinctive advantages of the proposed method, Table 9 compares it with recent representative dual-band filter designs.

It can be summarized that the proposed method is fully flexible with respect to the specifications of the two passbands, and the only limitation of the total and relative bandwidth (this last one, measured as the BWR) is technological, imposed by the materials and the manufacturing process: the minimum and maximum coupling. This is the case for both the coupling between SRRs (for the total, absolute bandwidth) and the coupling between the rings inside each SRR (for the relative bandwidth). The second advantage is that the design is divided into two steps, thanks to the frequency transformation. First, some filter elements are designed to achieve specific characteristics of the required bandpass response, including the band order and in-band reflection loss. Only then are the two passbands generated.

As a result, the two-stage procedure simplifies tuning, although the final response remains sensitive to narrow-gap and feed-line tolerances.

As shown in Table 9, the proposed method offers greater flexibility than recent dual-band filter design approaches. While previous works such as [1,3] offer compact structures and acceptable return loss, they generally rely on fixed or symmetric BWRs and limited coupling control. More advanced structures, such as those in [2,4], introduce complexity due to multilayer or CRLH-based implementations and still lack explicit BWR control.

In contrast, our approach provides full analytical control over the FBW and BWR of each passband. This is achieved through coupling-matrix synthesis combined with electromagnetic energy modeling of SRRs, enabling predictable, flexible design tuning. Additionally, the design process remains simple and compact, making it suitable for modern multiband applications without compromising performance.

The methods compared in Table 9 illustrate the trade-offs between tunability, design complexity, and performance in recent DB-BPFs. While solutions like [2,8] provide partial bandwidth control, they lack analytical frameworks for BWR control. Our method stands out by combining compact layout, full analytical synthesis, and high design simplicity.

### 3.3. Sensitivity to Fabrication Tolerances

To quantify the effect of fabrication tolerances on the proposed SRR-based filters, a combined geometrical sensitivity analysis was carried out for both prototypes by simultaneously perturbing the most critical dimensions of the structure by ±0.02 mm around their nominal values. In particular, the inter-ring gap, the feed-coupling gap, and the strip width were varied together in order to emulate a realistic fabrication deviation affecting the narrow coupling regions of the filters. For each perturbed case, the corresponding *S*-parameters were recomputed, and the main response quantities were extracted, namely the center frequencies of the two passbands, the 3 dB bandwidths, the return loss, and the BWR.

**Figure 11:**
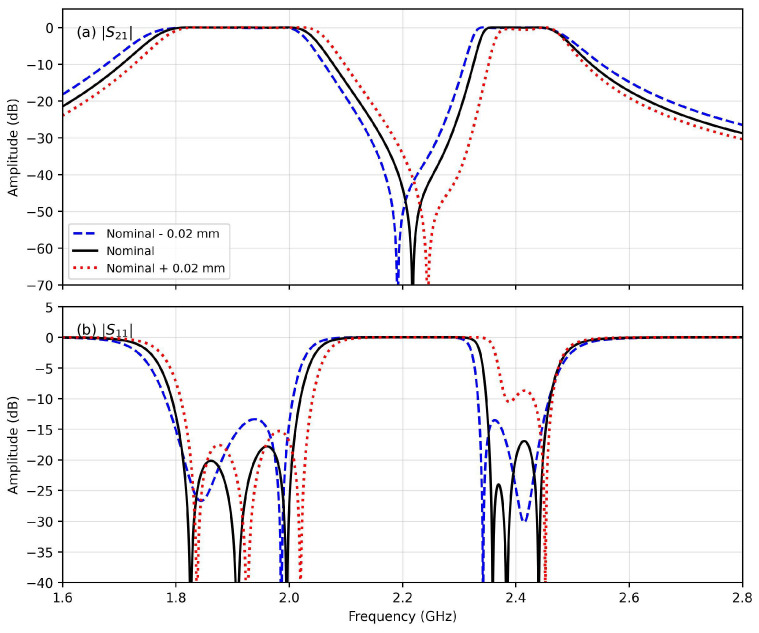
Combined geometrical tolerance analysis of the prototype with BWR 1:2. The figure shows the effect of simultaneous ±0.02 mm variations in the critical dimensions of the structure on the responses. (**a**) $|S_{21}|$ and (**b**) $|S_{11}|$ simulations analysis.

**Figure 12:**
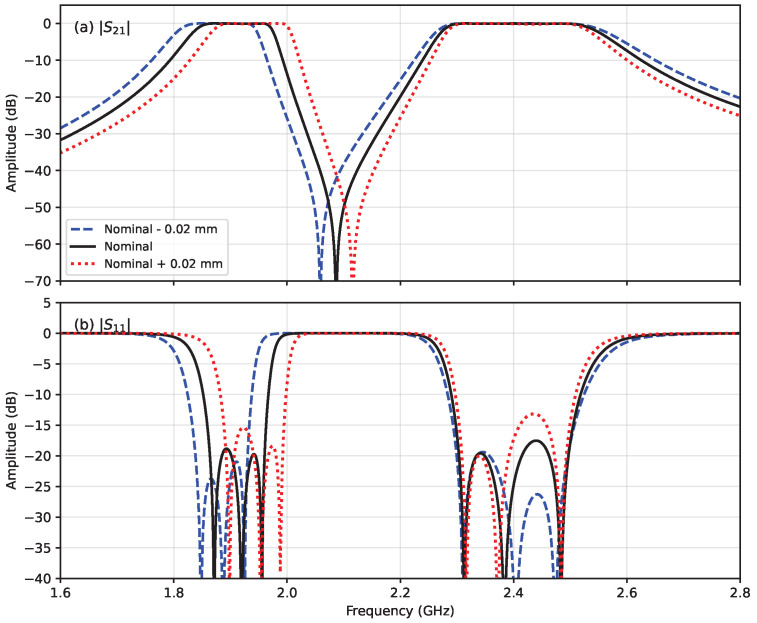
Combined geometrical tolerance analysis of the prototype with BWR 2:1. The figure shows the effect of simultaneous ±0.02 mm variations in the critical dimensions of the structure on (**a**) the transmission response $|S_{21}|$ and (**b**) the reflection response $|S_{11}|$.

Figure 11 and Figure 12, together with Table 10 and Table 11, summarize the effect of the combined geometrical perturbation on the prototypes with BWR 1:2 and BWR 2:1, respectively. For the BWR 1:2 prototype, the nominal response exhibits center frequencies of 1.908 GHz and 2.384 GHz, with bandwidths of 284 MHz and 146 MHz, giving BWR = 0.514. Under the −0.02 mm perturbation, the response shifts to 1.986 GHz and 2.342 GHz and the BWR increases to 0.581, whereas for the +0.02 mm perturbation the upper passband shifts upward to 2.452 GHz, its bandwidth decreases to 118 MHz, and the BWR is reduced to 0.414. For the BWR 2:1 prototype, the nominal case yields center frequencies of 1.920 GHz and 2.384 GHz, with bandwidths of 146 MHz and 288 MHz, giving BWR = 1.973. When the dimensions are simultaneously reduced by 0.02 mm, the BWR increases to 2.108, whereas for the +0.02 mm perturbation it decreases to 1.812. In both prototypes, the results show that simultaneous dimensional deviations produce measurable shifts in center frequency and bandwidth, and that the passband maintaining the asymmetric response is the most sensitive to the perturbation: the upper passband in the BWR 1:2 case and, again, the wider upper passband in the BWR 2:1 case. Overall, the combined tolerance analysis confirms that the final response of the proposed narrow-gap SRR-based structures remains sensitive to realistic manufacturing deviations. Since the three critical dimensions were perturbed simultaneously, the obtained results should be interpreted as the cumulative effect of fabrication tolerances rather than as the isolated contribution of a single geometrical parameter. These results therefore provide quantitative support for the tolerance discussion and reinforce the practical limitations associated with tightly coupled SRR implementations, particularly in the dimensions that control the asymmetric bandwidth response. That is consistent with broader reports showing that fabrication inaccuracies and material variations can shift the frequency response of microwave circuits, even though the exact mechanisms depend on the specific manufacturing technology. Ref. [20] showed systematic S-parameter frequency shifts in a fabricated microstrip filter due to inaccurate milling depth, and [21] described significant substrate-permittivity variation in 3D-printed microwave structures that required post-fabrication compensation.

The proposed synthesis methodology remains valid, since the target dual-band response can still be prescribed through the coupling-matrix-to-SRR design flow. However, the numerical tolerance analysis indicates that the final response is sensitive to geometric deviations in the structure’s strongly coupled regions. Consistent with the design formulation, the inter-ring spacing *s* is expected to be especially critical, since it governs the internal SRR coupling and directly affects the resonance splitting and the bandwidth ratio. The feed-coupling gap mainly influences the external coupling and, consequently, the passband matching and the realized bandwidths. The combined results obtained for the two prototypes confirm that even small simultaneous variations of ±0.02 mm already produce measurable shifts in center frequency and noticeable changes in the BWR. Therefore, the method should be regarded as analytically well-defined but is sensitive to fabrication tolerances in narrow-gap regions.

## 4. Conclusions

This work presented a synthesis and implementation method for compact dual-band bandpass filters based on split-ring resonators with controlled asymmetric bandwidth response. By combining coupling-matrix synthesis with an energy-based SRR model, the method enables explicit control of the center frequencies and the bandwidth ratio of the two passbands. Two fabricated prototypes validated the approach and showed good agreement between simulation and measurement. The results confirm that compact planar dual-band filters with asymmetric bandwidths can be designed without additional reconfigurable elements. All numerical and experimental demonstrations reported in this work correspond to implementations on Rogers RO3010; therefore, extension to a different substrate should be understood as requiring a new design optimization rather than a direct transfer of dimensions. The main contribution of this work is a methodology for precise, analytical control of asymmetric passbands using SRRs. The proposed architecture demonstrates competitive performance in insertion loss, return loss, and miniaturization, while maintaining design simplicity and adaptability across different communication standards. Future work will address tolerance sensitivity and reconfigurable extensions of the proposed topology.

Future work will conduct a deeper modal analysis of the proposed SRR topology, including current-density and electromagnetic-field distributions, to complement the present design-oriented methodology with a more detailed physical interpretation of the resonant mechanisms.

## Figures and Tables

**Table 1:** Specifications of the prototypes.

	Filter 1		Filter 2	
	Band 1	Band 2	Band 1	Band 2
Center freq. (GHz)	1.9	2.4	1.9	2.4
Bandwidth (MHz)	200	100	100	200
Return loss (dB)	19	19	19	19
BWR	0.5 (1:2)		2 (2:1)	

**Table 2:** Distributed primary parameters of the SRR sections.

BWR	1:2	2:1
$L_{l_{1}},\;L_{l_{2}}\;\left(\frac{\mu H}{m}\right)$	0.5379	0.5379
$L_{l_{c}}$, $\;\left(\frac{\mu H}{m}\right)$	$\left[\begin{array}{cc} 0.5161 & 0.1205\\ 0.1205 & 0.5161 \end{array}\right]$	$\left[\begin{array}{cc} 0.4547 & 0.0756\\ 0.0756 & 0.4547 \end{array}\right]$

**Table 3:** Design parameters of the BWR 1:2 resonators.

	First Resonance	Second Resonance
*Z*	$-j10\left[\begin{array}{cc} 1.448 & 1.433\\ 1.433 & 1.417 \end{array}\right]$	$-j10\left[\begin{array}{cc} -1.431 & 1.452\\ 1.452 & -1.473 \end{array}\right]$
$\left[\begin{array}{c} i_{1}\left(0\right)\\ i_{2}\left(l_{2}\right) \end{array}\right]$	$\left[\begin{array}{c} -0.7033\\ 0.7109 \end{array}\right]$	$\left[\begin{array}{c} 0.7122\\ 0.7020 \end{array}\right]$
$\left[\begin{array}{c} i_{1}\left(l_{1}\right)\\ i_{2}\left(l_{c}\right) \end{array}\right]$	$\left[\begin{array}{c} -0.7031\\ 0.7106 \end{array}\right]$	$\left[\begin{array}{c} 0.7118\\ 0.7014 \end{array}\right]$
$W_{M}$	1.3494	2.6889
$W_{M}$ ratio	0.5018	

**Table 4:** Simulated and measured results of the prototype with BWR 1:2.

	EM Simulations		Measurements	
Bands	1	2	1	2
Center freq. (GHz)	1.89	2.42	1.895	2.43
Bandwidth (MHz)	197	95	200	92
Return loss (dB)	19	19	16	18
Insertion loss (dB)	0.35	0.4	1.0	1.95
BWR	0.48		0.46	

**Table 5:** Design parameters of the BWR 2:1 resonators.

	First Resonance	Second Resonance
*Z*	$-j10\left[\begin{array}{cc} 0.353 & 0.887\\ 0.887 & 2.227 \end{array}\right]$	$-j10\left[\begin{array}{cc} -2.988 & 0.974\\ 0.974 & -0.318 \end{array}\right]$
$\left[\begin{array}{c} i_{1}\left(0\right)\\ i_{2}\left(l_{2}\right) \end{array}\right]$	$\left[\begin{array}{c} 0.9291\\ -0.3699 \end{array}\right]$	$\left[\begin{array}{c} 0.3099\\ 0.9508 \end{array}\right]$
$\left[\begin{array}{c} i_{1}\left(l_{1}\right)\\ i_{2}\left(l_{c}\right) \end{array}\right]$	$\left[\begin{array}{c} 0.8729\\ -0.3692 \end{array}\right]$	$\left[\begin{array}{c} 0.2743\\ 0.9476 \end{array}\right]$
$W_{M}$	2.6356	1.3212
$W_{M}$ ratio	1.9949	

**Table 6:** Simulated and measured results of the prototype with BWR 2:1.

	EM Simulations		Measurements	
Bands	1	2	1	2
Center freq. (GHz)	1.88	2.40	1.89	2.42
Bandwidth (MHz)	93	198	113	210
Return loss (dB)	18	17	11	13
Insertion loss (dB)	0.6	0.2	1.8	0.6
BWR	2.12		1.86	

**Table 7:** Comparison of reported unloaded quality factors $Q_{u}$ for representative resonators. The values correspond to different substrates, thicknesses, and technologies; therefore, the comparison is qualitative rather than substrate-normalized.

	$Q_{u}$	
Resonators	Lower	Upper
Loop resonator [4]	85	90
Stub-loaded ring [1]	88	95
CRLH-TL resonator [2]	90	92
Modified open-loop [3]	91	94
NB-SRR (as in [14])	90	91
Modified hexagonal SRR [18]	87	90
BWR 1:2 (proposed model)	91	94
BWR 2:1 (proposed model)	91	95

**Table 8:** Expanded comparison of representative dual-band bandpass filters and the proposed designs. NR: not reported in the present manuscript comparison.

Refs.	Substrate	Order	${\mathrm{FBW}}_{1}/{\mathrm{FBW}}_{2}$	Insertion Loss	Return Loss	Inter-Band Att.	Normalized Size	Extra Tuning/Reconfig.	Explicit BWR
				(dB)	(dB)	(dB)	$\left(\lambda_{g}\times \lambda_{g}\right)$	Elements	in Synthesis
[1]	NR	NR	6.1/4.8	1.2/1.8	NR	50	0.38 × 0.22	NR	No
[2]	NR	NR	8.0/3.2	1.3/1.5	NR	40	0.45 × 0.28	NR	No
[3]	NR	NR	10.0/5.1	1.0/1.6	NR	52	0.30 × 0.20	NR	No
[4]	NR	NR	5.6/7.5	1.1/1.4	NR	48	0.34 × 0.19	NR	No
[5]	Taconic RF-60A	4/5	39/20	0.2/0.2	NR	NR	NR	No	No
[6]	RT/Duroid 5870	NR	72/2.6	<1/<1.5	>10/>10	NR	0.58 × 0.122	No	No
[7]	Rogers Duroid 5880	NR	2.12/1.15	0.5/0.86	17.56/17.9	24	0.23 × 0.11	No	No
[14]	NR	NR	6.5/6.5	1.5/1.9	NR	42	0.36 × 0.25	No	No
BWR 1:2	Rogers RO3010	3	10.6/3.8	1.0/1.95	16/18	48	0.60 × 0.20	No	Yes
BWR 2:1	Rogers RO3010	3	6.0/8.7	1.8/0.6	11/13	54	0.54 × 0.20	No	Yes

**Table 9:** Comparison of dual-band filter bandwidth-control capability and implementation requirements. FBW control level: 0 = fixed; 1 = limited/adjustable (range-tuning without independent specification of both bands); 2 = independently specifiable FBW1 and FBW2 by design procedure. BWR control level: 0 = not addressed; 1 = constrained (fixed ratio/symmetric bands/partial); 2 = explicitly specifiable BWR as a design parameter.

Refs.	Method Used	FBW Control Level (0–2)	BWR Control Level (0–2)	Explicit BWR Parameter in Synthesis (Yes/No)	Add. Tuning/ Reconf. Elements Required (Yes/No/NR)
[1]	Stub-loaded resonators	1	1	No	NR
[2]	CRLH-TL with coupled lines	1	0	No	NR
[3]	Miniaturized dual-band BPF with multiple TZs	0	1	No	NR
[4]	In-line dual-band BPF with multiple TZs	0	1	No	NR
[5]	Element-by-element dual-band synthesis with TSSIRs and SIIs	0	0	No	No
[6]	A-SIR and metacell asymmetric dual-band filter	1	1	No	No
[8]	Folded stepped-impedance resonator	1	1	No	NR
This work	Energy-based SRRs + coupling-matrix Synthesis	2	2	Yes	No

**Table 10:** Extracted response quantities under combined geometrical perturbations of ±0.02 mm around the nominal design for the prototype with BWR 1:2.

Case	$f_{01}$ (GHz)	${\mathrm{BW}}_{1}$ (MHz)	$f_{02}$ (GHz)	${\mathrm{BW}}_{2}$ (MHz)	BWR
Nominal − 0.02 mm	1.986	289	2.342	168	0.581
Nominal	1.908	284	2.384	146	0.514
Nominal + 0.02 mm	1.926	285	2.452	118	0.414

**Table 11:** Extracted response quantities under combined geometrical perturbations of ±0.02 mm around the nominal design for the prototype with BWR 2:1.

Case	$f_{01}$ (GHz)	${\mathrm{BW}}_{1}$ (MHz)	$f_{02}$ (GHz)	${\mathrm{BW}}_{2}$ (MHz)	BWR
Nominal − 0.02 mm	1.925	148	2.404	312	2.108
Nominal	1.920	146	2.384	288	1.973
Nominal + 0.02 mm	1.954	144	2.372	261	1.812

## Data Availability

The original contributions presented in this study are included in the article. Further inquiries can be directed to the corresponding author.

## Supplementary Materials

The following supporting information can be downloaded at: https://www.mdpi.com/article/10.3390/s26113519/s1, Figure S1: Fabricated prototype with BWR 2:1 used for the experimental validation. Figure S2: Representative raw VNA screenshot of the measured response of the fabricated prototype with BWR 2:1. Figure S3: Fabricated prototype with BWR 1:2 used for the experimental validation. Figure S4: Representative raw VNA screenshot of the measured response of the fabricated prototype with BWR 1:2.

## Abbreviations

The following abbreviations are used in this manuscript:

SRR	Split ring resonator
BPFs	Bandpass filters
CRLH	Composite right/left-handed
TL	Transmission lines
FBW	Fractional bandwidth
DSRR	Defected split-ring resonator
SIR	Stepped-impedance resonator
BWR	Bandwidth ratio
EM	Electromagnetic
