# Supplementary material for: Dual Band-Pass Filter Based on Split Ring Resonators with Controlled Asymmetric Bandwidth Response
Source: Sensors (Basel). 2026 Jun 2;26(11):3519. doi: 10.3390/s26113519 (PMC13259517; doi:10.3390/s26113519)
Supplement: Supplementary file 1 [file sensors-26-03519-s001.zip › sensors-4252977-supplementary.pdf]

Supplementary Material

The supplementary files include representative screenshots of the raw vector network analyzer (VNA) measurements and the exported measured S_11 and S_21 traces for both fabricated prototypes. These materials are provided to complement the experimental validation reported in the main manuscript by showing the original instrument visualization and the corresponding measured responses used for comparison with the lossy HFSS simulations and the circuit model.

BWR 2:1

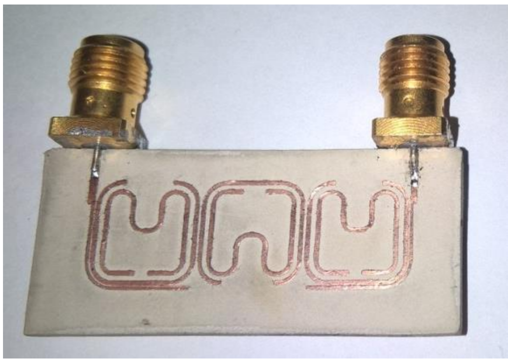

Figure S1:Fabricated prototype with BWR 2:1 used for the experimental validation.

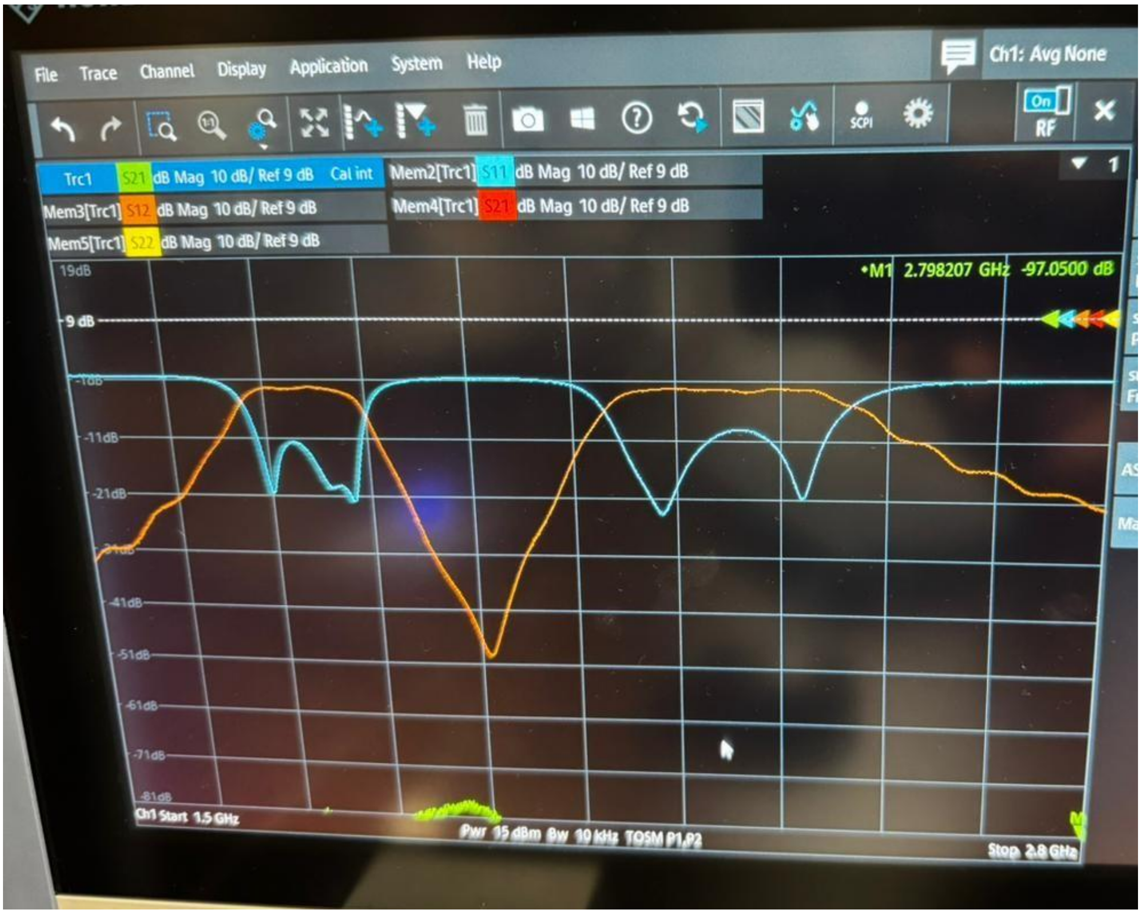

Figure S2. Representative raw VNA screenshot of the measured response of the fabricated prototype with BWR 2:1.

BWR1:2

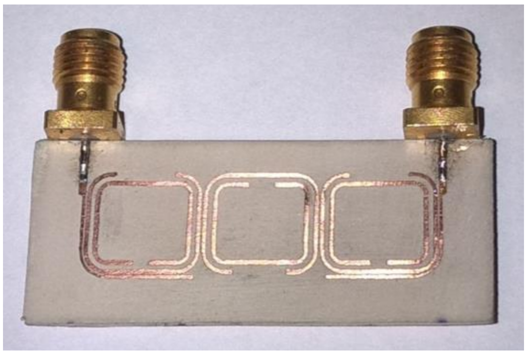

Figure S3: Fabricated prototype with BWR 1:2 used for the experimental validation.

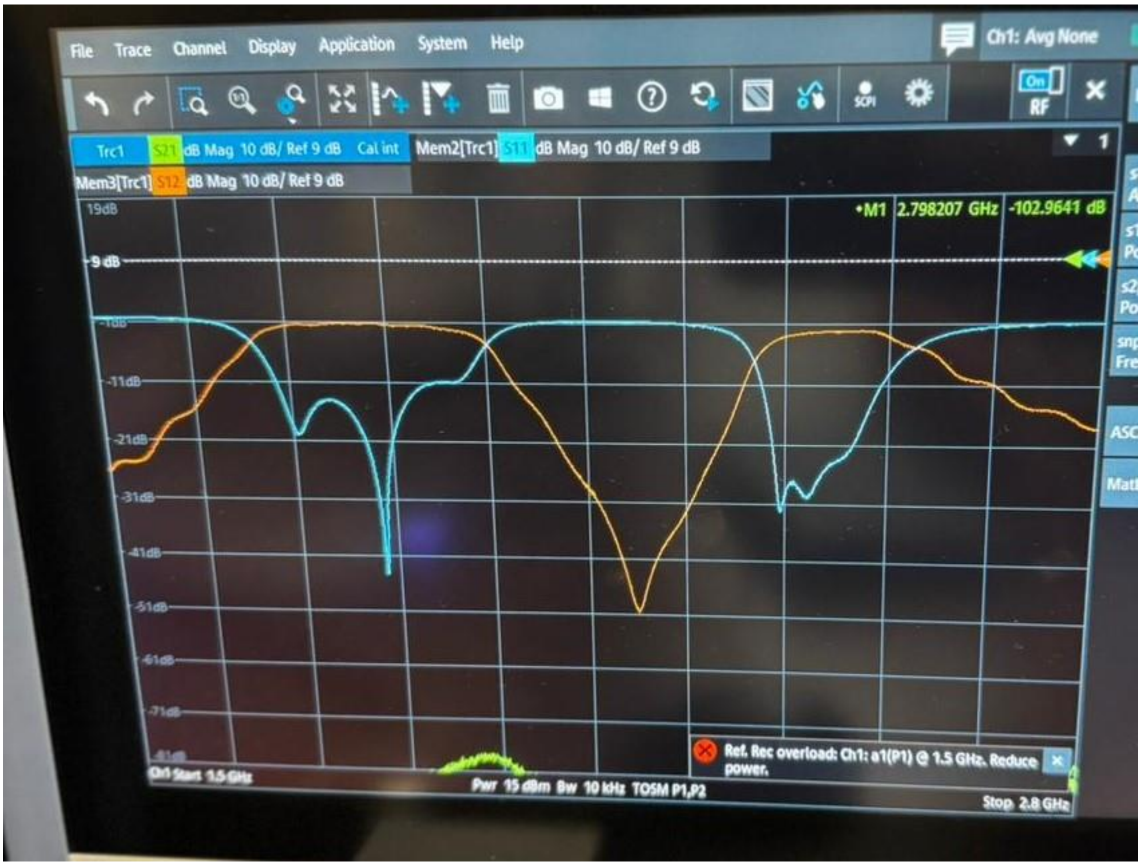

Figure S4: Representative raw VNA screenshot of the measured response of the fabricated prototype with BWR 1:2.
